# Structural family factors and bullying at school: a large scale investigation based on a Chinese adolescent sample

**DOI:** 10.1186/s12889-021-12367-3

**Published:** 2021-12-11

**Authors:** Haoran Wang, Yuanyuan Wang, Guosheng Wang, Amanda Wilson, Tingting Jin, Longjun Zhu, Renjie Yu, Shuilan Wang, Weijia Yin, Huihui Song, Shun Li, Qiufang Jia, Xiaobin Zhang, Yong Yang

**Affiliations:** 1 Dalian Seventh People’s Hospital, 179# Lingshui Road, Dalian, 116023 China; 2 Division of Psychology, Faculty of Health and Life Sciences, De Montfort University, Leicester, UK; 3 Suzhou Guangji Hospital, 11# Guangqian Road, Suzhou, 215137 China; 4 Suzhou No.1 High School of Jiangsu Province, , 279# Gongyuan Road, Suzhou, 215011 China

**Keywords:** Adolescents, Bullying, Socio-economic status

## Abstract

**Backgrounds:**

Various family factors have been identified in association with school bullying and the involvement of children and adolescents in bullying behaviors.

**Methods:**

A total of 11,919 participants (female = 6671, mean age = 15) from 22 middle schools in Suzhou City, China completed the questionnaire. The associations between structural family factors (family socio-economic status, living arrangement, number of siblings, whether they were local residents/migrants, had an urban/rural *hukou [a household registration system in China]*, parental and maternal education levels, and other various bullying-related constructs (i.e. bullying witnessing, bullying involvement, bystander intervention, and fear of being bullied) were all examined. Odds ratios (ORs) adjusted for covariates were calculated for the four bullying-related constructs (bullying witness, bullying involvement, bystander intervention, and reactions to being bullied) using structural family factors.

**Results:**

The result showed that all demographic household characteristics were associated with bullying at school except for being from a single-child family. Adolescents from rural families witnessed more bullying incidents than those from local families (OR = 1.35, 95% CI: [1.09, 1.68]). Adolescents who come from migrant families (OR = 1.12, 95% CI: [1.07, 1.43]) with a rural hukou (OR = 1.31, 95% CI: [1.00, 1.74]) and low parental education levels (OR = 1.42, 95% CI: [1.01, 2.57]) were more likely to be bullies. Adolescents who came from migrant families (OR = 1.37, 95% CI: [1.03, 1.82]), with low maternal education levels (OR = 1.42, 95% CI: [1.06, 1.91]) engaged in more negative bystander intervention behaviors. Furthermore, adolescents with less educated mothers experienced a higher fear of being bullied (*never* versus *sometimes*: OR = 1.33, 95% CI: [1.00, 1.85]; *never* versus *usually* OR = 1.39, 95% CI: [1.01, 1.20]).

**Conclusions:**

A systematic examination of the relationship between school bullying and demographic household characteristics may be used to inform school policies on bullying, such as training management on the importance of paying attention to adolescents from disadvantage household backgrounds. Identifying demographic factors that may predict bullying can also be used to prevent individuals from becoming involved in bullying and reduce the related negative consequences from being bullied.

**Supplementary Information:**

The online version contains supplementary material available at 10.1186/s12889-021-12367-3.

## Introduction

School bullying has been widely identified as a risk factor for adolescents’ poor psychological wellbeing and a major challenge for school management. It is estimated that the percentage of students involved in bullying ranges from 8 to 40% [1]. In China this number is estimated to be nearly 20% [2]. Considering the pervasive and detrimental effects of bullying on the psycho-social development of adolescents [3], it is necessary to further explore both the risk and protective factors of school bullying. Previous literature has showed that socio-demographic factors, including sex, race, age, family socio-economic status etc. may influence the risk of bullying on campus [4].

Family systems have long been recognized as a source of influence on adolescents’ social behaviors at school, including aggressive behaviors like bullying [5]. Previous studies have shown that several family characteristics are closely related to school bullying, such as family environments, parent-child relations, and family norms [6]. Family environments have been conceptualized as a multi-component factor including general feelings of safety at home, perceived parental support, and so forth [7]. Previous research has further found that negative family environments may be associated with higher risks of bullying victimization at school [7]. Oliveira and colleagues [8] found that positive family interactions may protect adolescents from being involved in school bullying (both as perpetrators and victims). Evidence also shows that adolescents who have good relationships with their parents are less likely to be bullies or victims [9]. Also, Orozco-Vargas [10] found that family moral values were closely related to the likelihood of being bullied among adolescent girls.

Living in a multi-generation family could also be associated with bulling at school. Research has shown that grandparents’ involvement in the family education could be associated with less adjustment problems for adolescents [11]. Zhang [12] found that co-habiting with grandparents could significantly facilitate adolescents’ academic performance, which is also a well-established protective factor that buffers against involvement in school bullying. Considering the positive effects of grandparents’ involvement, further studies are needed to examine the protective effects of living with grandparents on involvement into school bullying.

Having siblings has also been identified as a protective factor for school bullying. Living in a single child family could significantly influence adolescents’ social adjustment at school [13], which is closely related to school bullying [14]. However, such influence is complex. On the one hand, single-child families are prone to overly attending to the only child, which could hinder normal social development and lead to maladjustments, such as peer conflicts and loneliness [15]. On the other hand, single-child families are able to allocate more resources to the only child and thus could enhance the general well-being of the child (Yang J: Has the one-child policy improved adolescents educational wellbeing in China?, Unpublished).

Besides, there are various other household demographic characteristics, such as family structures and socio-economic status that could also be related to school bullying [16]. For example, Ackerman and colleagues [17] found that adolescents from unmarried families or co-habiting families are more likely to engage in delinquent and aggressive behaviors, such as bullying. Similarly, Fung and colleagues [18] found that adolescents from single-mother or step-mother families engaged in more aggressive behaviors compared to their counterparts. Previous evidence also showed that adolescents from a disadvantageous socio-economic background (e.g. lower household incomes, lower parental/maternal education level, and living in rural areas) scored higher on traits like impulsivity and showed more anti-social behaviors than their socio-economically advantaged counterparts [19]. However, more recent evidence suggests that family characteristics do not act as predictors to involvement in school bullying [20]. These inconsistent findings about the effects of household characteristics on bullying warrant further investigation.

Moreover, the relationships between household demographics, Chinese culture, and bullying have had little exploration. For example, *single child family* is defined as a family composed of one child [21]. As the “one-child” policy was enforced in China since 1979, being a single-child family is predominant in urban areas of China [21]. It is important to note that China now operates under a ‘two-child’ policy since 2015 [22]. While the current urban families of China is skewed towards having an only child, it is likely that in the future, due to the two and three child policy changes, that there will be a more diverse range of family compositions in China. *Internal migrant family* is defined as rural households who have moved to Chinese cities [23]. Due to large-scale migration of Chinese families from rural areas to cities in the last two decades, *internal migrant families* are much more prevalent in China than in Western countries [23]. This suggests that migration is then an important socio-demographic characteristic in the Chinese context, and thus Western research cannot be applied to China without adaption due to the difference in the number of internal migrants. *Multi-generation family* refers to families that consist of more than two generations in one household. Again it is more prevalent in China than in Western countries as part of China’s cultural traditions [24]. It is therefore necessary to examine whether adolescents from these types of families are more or less likely to be involved in school bullying. Previous evidence showed that living in an internal migrant family could be associated with the probability of adolescents’ involvement in bullying. One recent investigation [25] showed that adolescents from such families are more likely to perpetrate bullying than their local counterparts. However, there is also research evidence [26] suggests that internal migrated adolescents did not experience more peer problems than non-migrant adolescents. Again, considering such inconsistencies in the literature, whether being a single child and living in an internal migrant family and how this could be related to adolescents’ bullying experience at school should be further explored.

Taking into consideration the contradicting findings above and the need for examining school bullying in the Chinese cultural context, there are several gaps in the literature that this study aims to address. First, the relationships between some Chinese household types (e.g. single-child family, intergenerational family, internal migrated family) and bullying have rarely been explored in research. Second, most of the studies focus only on the association between family structures and the roles in bullying (i.e. bully, victim, and bully/victim), which neglects other bullying-related constructs [27]. In order to fill this gap in the literature, the current research focused on the association between household demographic characteristics and a full range of bullying indicators including: *bullying witnessing*, *bullying involvement*, *bystander intervention*, and *reactions to being bullied*. The researchers hypothesized that: (1) low paternal/maternal education levels and holding a rural *hukou* would be associated with a higher risk of being bullied or bullying others at school; (2) living in a multigenerational family could be associated with a lower risk of being bullied or bullying others at school. Associations between other household family demographics and bullying-related constructs could not be predicted due to the paucity of research evidence so far.

## Methods

### Participants

In January 2019, 22 middle schools (grades 7–11) in Suzhou, a major city in Eastern China, were invited to participate in the research, with no school declining the invitation to participate. Cluster sampling methods were used to select middle and high schools in one of the districts in Suzhou City. A total of 11,919 questionnaires were returned with the response rate being 83.2%. Assent was obtained from participates and passive informed consent was obtained from their main guardian prior to the pencil-and-paper questionnaires being filled in at school. Teachers were involved in obtaining passive parental consents and an information sheet was given to parents/guardians allowing them the opportunity to consider whether their child should take part in the study and providing them the opportunity to inform the teacher if they did not want their child to participate. If the parent did not inform the teacher that they objected to the research, it was passively assumed that the teacher had their consent for the child to participate (Hollmann & McNamara, 1999). A non-anonymous survey format was adopted in the current research. Participants were instructed to sign their name on the questionnaire and were assured that their name would be kept confidentially. The data inputted was anonymous to the research team. The research was approved by the Ethics Committee of the Mental Health Center of Suzhou (approval SGLS2017–037).

### Measures

#### Social demographics checklist

The socio-demographic collected included: (1) sex and age; (2) rural/urban hukou; (3) migrant status (local residence/moved from other areas of China); (4) living arrangement (living with parents/grandparents/other relatives); (5) education level of parents; (6) family economic status. Considering the difficulty for adolescents to report the household income precisely, we used an item “are you living in your own house or in a rented house?” to roughly measure their family economic status; and finally we collected information on (7) being a single child or having sibling(s).

#### Bullying questionnaire

Items in the bullying questionnaire include: (1) Bullying Witness: “During this school year how often have you seen someone being bullied?” (2) Bullying Involvement: “During this school year how often have you been bullied at school?” and “During this school year how often have you bullied others?” (3) Bystander Intervention: “If you saw bullying at school, what would you do?”, bystander reactions were classified as not-intervening (i.e. look on and do nothing), negatively intervening (teasing those who were being bullied), and positively intervening (helping those who were being bullied). (4) Fear of Being Bullied: “During the past year how often did you miss school because you felt unsafe, uncomfortable, or nervous at school or on your way to/from school?” In the current research, two traditional forms of bullying, verbal insult (i.e. teasing) and physical assaults (i.e. pushing, shoving, kicking, slapping or hitting) were measured [28].

### Statistical analysis

All statistical analyses were conducted using SPSS 26.0 and Mplus 7.0. Considering the hierarchical nature of the data (i.e. individuals nested into schools), multilevel regression modeling was used to take possible clustering effects into consideration. First, intra-class correlations (ICCs) were calculated to determine the degree of homogeneity of the outcome variables within the clusters [29]. Then, the two-level logistic regression models with random intercepts [30] were estimated and multivariate-adjusted odds ratios (ORs) for the four bullying-related constructs (bullying witness, bullying involvement, bystander intervention, and reactions to being bullied) were calculated with the various socio-demographic characteristics of family households.

As bullying witnessing and fear of being bullied were coded as an ordinal categorical variable (never, sometimes, usually, and almost every day), a parallel line test was conducted to examine whether the associations between predictive and outcome variables were different across categories.

## Results

### Preliminary results

Sample characteristics are shown in Table 1. Results showed that 26.4% of boys and 14.3% of girls had been involved in bullying at least once in this study and all participants were classified in four categories using a dichotomous response of yes or no to each of the four categories: bullies (*n* = 1515, 12.7%), victims (*n* = 463, 3.9%), bully/victim (*n* = 480, 4.0%), and participants not involved in bullying (*n* = 9461, 79.4%).

**Table 1:** Characteristic of the participants

Demographic information	N (%)
Sex	
Female	5486 (46.02%)
Male	6433 (53.98%)
Age (Means and Standard Deviations.)	15.0 (1.47)
Migrant status	
Local	4832 (37.71%)
Migrant from other areas of China	4495 (40.54%)
Hukou status	
Urban hukou	6735 (56.50%)
Rural hukou	4399 (36.91%)
Living arrangement	
Living with parents	11,206 (94.01%)
Living with grandparents	369 (3.09%)
Living with other relatives	208 (2.90%)
Single child or not	
Being the single child	7666 (64.31%)
Not being the single child	4006 (34.4%)
Paternal education level	
Middle school or lower	5778 (48.8%)
Senior high or vocational school	3672 (31.0%)
Bachelors or higher	2394(20.2%)
Maternal education level	
Middle school or lower	6650 (56.2%)
Senior high or vocational school	3126 (26.4%)
Bachelors or higher	2067 (17.5%)

Results showed that ICCs for all four dependent variables (bullying witnessing, bullying involvement, fear of being bullied, bullying intervention) ranged from 0.020 to 0.043, which means that 2 to 4.3% of the variation in the dependent variables could be attributed to the variation of level-2 variable (i.e. school).

### Family characteristics and bullying witnessing

Odds ratios (ORs) for bullying witnessing are listed in Table 2. A parallel line test showed that the associations between family characteristics and witnessing bullying were not different across categories (*p* = 0.51). Results showed that adolescents who came from rural families witnessed more bullying scenarios than those who came from urban families (*never* versus *often* OR = 1.35, 95% CI: [1.09, 1.68]). Other family demographics (family status, economic status, etc.) were not related to witnessing bullying.

**Table 2:** Odds ratios for bullying witnessing

	Bullying witnessing (never versus sometimes)			Bullying witnessing (never versus often)		
	Odds Ratios	S.E.	95% CI	Odds Ratios	S.E.	95% CI
Intercepts	.29*	.23	(.18, .46)	.11*	.35	(.06, .22)
Single child or not						
No	.93	.06	(.89,1.12)	1.15	.09	(.92,1.35)
Yes (reference)						
Local residents or not						
No	.99	.06	(.89, 1.12)	.1.13	.09	(.93, 1.37)
Yes (reference)						
Family economic status						
Living in a rented house	.94	.05	(.81,.1.09)	.84	.12	(.66,1.07)
Living in an owned house (reference)						
Living arrangement						
Living with grandparents	1.35	.19	(.93, 1.96)	.99	.28	(.56,1.74)
Living with parents (reference)						
Urban/rural hukou						
Rural	1.02	.07	(.88.1.16)	1.35*	**.12**	**(1.09,1.68)**
Urban (reference)						
Parental education level						
Middle school or lower	1.08	.09	(.83,1.42)	1.20	.08	(.94, 1.41)
Bachelor’s or higher (reference)						
Maternal education level						
Middle school or lower	.86	.08	(.72,1.02)	.87	.02	(.66,1.15)
Bachelor’s or higher (reference)						

(1) bullying witnessing is coded as an ordinal categorical variable (*never, sometimes, usually*) and *never* group was chosen as the reference; (3) *p < 0.05, **p < 0.01; ***p < 0.001

### Family characteristics and bullying involvement

Adjusted ORs for bullying involvement are listed in Table 3. Results showed that adolescents who come from migrant families (OR = 1.12, 95% CI: [1.07, 1.43]) with a rural hukou (OR = 1.31, 95% CI: [1.00, 1.74]), and low parental education levels (OR = 1.42, 95% CI: [1.01, 2.57]) were more likely to be bullies. Coming from migrant families was the only factor that was related to the higher likelihood of being a bully/victim (OR = 1.23, 95% CI: [1.06, 1.43]). No factors in the current research were associated with the likelihood to be a victim.

**Table 3:** Odds ratios for bullying involvements

	Victimization			Perpetration			Perpetration- victimization		
	OR	SE	95%CI	OR	SE	95%CI	OR	SE	95%CI
Intercept	.14**	.18	(.11, .17)	.29**	.45	(.19, .44)	.06**	.28	(.42, .87)
Single child or not									
No	.84	.12	(.66,1.08)	1.12	.10	(.86,1.45)	.95	.12	(.82, 1.09)
Yes (reference)									
Local residents or not									
No	.99	.13	(.77,1.28)	1.12*	**.11**	**(1.07,1.43)**	1.23*	**.13**	**(1.06,1.43)**
Yes (reference)									
Family economic status									
Living in a rented house	1.04	.16	(.75, 1.43)	.92	.12	(.67,1.21)	.95	.07	(.79,1.14)
Living in an owned house (reference)									
Living arrangement									
Living with grandparents	.55	.17	(.30, 1.02)	.75	.18	(.37, 1.49)	1.02	.20	(.64, 1.60)
Living with parents (reference)									
Urban/rural hukou									
Rural	1.17	.09	(.88,1.56)	1.31*	**.13**	**(1.00,1.74)**	.92	.12	(.78. 1.09)
Urban (reference)									
Parental education level									
Middle school or lower	1.13	.17	(.80, 1.59)	1.42*	**.17**	**(1.01, 2.57)**	1.16	.24	(.94, 1.43)
Bachelor’s or higher (reference)									
Maternal education level									
Middle school or lower	.75	.17	(.53, 1.07)	1.18	.19	(.79, 1.77)	1.01	.18	(.76, 1.83)
Bachelor’s or higher (reference)									

(1) bullying involvement was divided into four categories, and the category for reference is “not-involved”; (2) *p < 0.05, **p < 0.01; ***p < 0.001

### Family characteristics and bystander intervention

ORs for bystander intervention are listed in Table 4. Results showed that adolescents who came from migrant families (OR = 1.37, 95% CI: [1.03, 1.82]) with low maternal education levels (OR = 1.42, 95% CI: [1.06, 1.91]) engaged in more negative intervention based behaviors such as teasing those who were bullied. Other family factors (family status, economic status, etc.) were not associated with bullying intervention.

**Table 4:** Odds ratios for bystander intervention

	Negatively intervening			Positively intervening		
	OR	SE	95%CI	OR	SE	95%CI
Intercept	.35*	.32	(.61, 1.34)	.28*	.28	(.15, .51)
Single child or not						
No	.1.02	.05	(.83, 1.26)	1.05	.09	(.73,1.51)
Yes (reference)						
Local residence or not						
No	1.37*	**.07**	**(1.03, 1.82)**	.91	.10	(.82, 1.33)
Yes (reference)						
Habiting status						
Living in a renting house	1,01	.05	(.81,.1.25)	1.01	.09	(.62, .1.64)
Living in an owned house (reference)						
Living arrangement						
Living with grandparents	1.52	.04	(.93, 1.89)	.83	.12	(.32,.2.15)
Living with parents (reference)						
Urban/rural						
Rural	.98	.06	(.77,1.24)	.1.28	.24	(.88, .1.86)
Urban (reference)						
Parental education level						
Middle school or lower	.93	.06	(.70,1.24)	1.05	.09	(,61 1.64)
Bachelor’s or higher (reference)						
Maternal education level						
Middle school or lower	1.42*	**.12**	**(1.06,1.91)**	1.08	.14	(.65, 1.80)
Bachelor’s or higher (reference)						

*p < 0.05, **p < 0.01; ***p < 0.001

### Family characteristics and fear of being bullied

Adjusted ORs for fear of being bullied are listed in Table 5. A Parallel line test showed that the associations between family factors and witnessing bullying were not different across categories (*p* = 0.66). Results showed that adolescents with a less educated mother suffered higher levels of fear of being bullied (*never* versus *sometimes*: OR = 1.33, 95% CI: [1.00, 1.85]; *never* versus *usually* OR = 1.39, 95% CI: [1.01, 1.20]). Other family factors were not associated with fear of being bullied in the current study.

**Table 5:** Odds ratios for reactions to being bullied (fear of being bullied)

	Fear of being bullied (never versus sometimes)			Fear of being bullied (never versus usually)		
	Odds Ratios (ORs)	Standard Error	95% CI	Odds Ratios (ORs)	Standard Error	95% CI
Intercepts	.09**	.28	(.01, .72)	.07**	.35	(.04, .15)
Single child or not						
No	1.06	.08	(.86,1.32)	.64	.12	(.41, .99)
Yes (reference)						
Local residence or not						
No	1.86*	.08	(1.09,1.97)	1.46	.13	(.92, 2.32)
Yes (reference)						
Habiting status						
Living in a renting house	.83	.09	(.63,1.08)	.89	.08	(.53, 1.56)
Living in an owned house (reference)						
Living arrangement						
Living with grandparents	.79	.21	(.44,1.41)	1.97	.24	(.27, 2.27)
Living with parents (reference)						
Urban/rural						
Rural	1.04	.09	(.75,1.26)	.74	.14	(.48, 1.15)
Urban (reference)						
Parental education level						
Middle school or lower	.86	.11	(.65, 1.16)	.92	.14	(.48, 1.71)
Bachelor’s or higher (reference)						
Maternal education level						
Middle school or lower	1.33*	**.12**	**(1.00,1.87)**	1.39*	**.11**	**(1.01, 1.99)**
Bachelor’s or higher (reference)						

(1) fear of being bullied is coded as an ordinal categorical variable (never, sometimes, usually) and the never group was chosen as reference; (2) *p < 0.05, **p < 0.01; ***p < 0.001

## Discussion

In the current research, the associations between household socio-demographic characteristics including family migrant status (migrants/non-migrants), hukou status (urban/rural hukou), parental/maternal education level, and living arrangements were explored in relation to four bullying-related constructs. Results indicated that the aforementioned demographic factors were closely related to adolescents’ bullying behaviors at school. To the best of our knowledge, this is the first research study to systematically examining the relations between family demographics and bullying-related constructs.

This study found that migrant status (migrants/local residents) was the only sociodemographic factor associated with bullying witnessing, with adolescents from migrant families observing more incidents of bullying at school. Previous research has also shown that migrant adolescents do not have equal access to various social welfare services (i.e. entrance to public schools and health care) compared to their urban counterparts, and they are also more likely to experience peer exclusion or discrimination [31]. Previous evidence showed that individuals who witnessed bullying scenarios were more likely to get involved in bullying than counterparts [32]. Together with the current results, it could be suggested that the disadvantages faced by migrant adolescents may render them more susceptible to school bullying. Further research is required to explore the impact of migrant status on bullying in schools to better understand this finding.

As hypothesized, being migrants, holding rural hukou, and low parental education levels were positively associated with the risk of being bullies (or bully/victims). This result is consistent with previous findings which show that having a low household income and low parental social statuses are predictive of delinquency during adolescence [33]. Migrant adolescents may hold different social norms than their local counterparts, and intolerance to customary differences may instigate a bully environment in schools. Research further shows that bullying behaviors may have different implications for rural and urban adolescents. As shown in the current study and several previous studies, a large proportion of internal-migrants in China are from rural areas [23]. Because of this, there is a noticeable difference in bullying perception between rural and urban adolescent. A large proportion of rural adolescents (especially males) regard bullying others as a status symbol (i.e. masculine capital), while urban adolescents usually interpret bullying as an act of “lack of self-restraint” and “rudeness” [34]. This differentiation in social values may also explain why migrant adolescents were more likely to be bullies in this study. In addition, adolescents who have a less educated father (but not mother) were also more likely to be bullies in this study. Parental education level has long been regarded as an important socio-economic factor, considering the significant effect of socio-economic characteristics on adolescents’ social behaviors; the association between fathers’ education level and bullying may partly be due to fathers’ dominant role in maintaining the family’s socio-economic status, which the child mimics [35].

Contrary to our original hypothesis, adolescents who lived with their grandparents did not have a higher or lower likelihood of being bullies. This result is inconsistent with some of the previous research that suggests cohabitating with grandparents could be positively associated with adolescents’ social adjustments. Previous research [11] evaluated how grandparents’ involvement, but not *living only with grandparents,* could affect the possibility of involvements in bullying for adolescents. However, the family dynamics could be quite different when grandparents’ are only involved with the child’s upbringing, but not living with the child. The child living only with their grandparents could possibly explain the inconsistency presented in research results. Future studies are required to clarify the role of grandparents in preventing or exacerbating bullying at school, with special care paid to exploring the differences in family composition (living with grandparents and parents, living *only* with grandparents, living with parents and receiving care from grandparents).

It is important to note that all the aforementioned family demographic characteristics were not associated with a high risk of being victims of bullying. Bullies (or bully/victims) are different from victims in terms of behavioral patterns. Bullying perpetration (or perpetration-victimization) has been conceptualized as “being proactively aggressive”, referring to “… cold blooded and goal-directed bullying behaviors” [36]. Looking at the current results of this study, adolescents from disadvantageous families had a higher risk of being proactive rather than reactive bullies. Further research is needed to explore the different relations between family demographics and proactive or reactive bullying at school.

In our study, adolescents from migrant families also had a higher likelihood of negatively reacting to those being bullied (e.g. teasing). According to previous research [37], adolescents with better social skills (such as high empathy, high self-control etc.) are more likely to intervene and provide help when they witness bullying scenarios. Therefore, having better social skills might explain the association between family economic status and intervention as a bystander. The results from our study are consistent with previous findings that adolescents from low economic backgrounds have a higher rate of delinquency [35]. Adolescents then with highly-educated parents are less likely to experience negative emotions (i.e. fear of school) than their counterparts. This indicated that both parental and maternal education levels could be a protective factor against bullying.

There are several limitations to the current research. First, all participants were from Suzhou, an economically advantageous city in Eastern China. Considering the main focus is on socio-demographic factors, such sampling methods may render the results prone to selection bias. Second, considering the cross-sectional nature of the current research, only correlations between bullying and the demographic factors could be explored. Longitudinal design should be adopted in future studies to examine causal relationships between the two constructs. Third, the measurements of some bullying-related constructs were simplified for concision. For example, only two forms of bullying (i.e. verbal/physical) were measured in the current research, with other forms, such as cyber-bullying and social isolation being unexamined. Also, the widely recognized features of school bullying (such as intention and power imbalances) were not considered appropriately in the current research. Participants may misunderstand the complex concept of bullying. For example, they could possibly regard some conflicts without power imbalances (e.g. arguments or fights) as bullying behaviors, which is not the aim of the current research. Future studies should explore how family demographic factors relate to a wide range of different forms of bullying, with a more accurate definition. Considering the complexity of behavioral patterns of bystanders, the current categorization (non-reaction, positively intervening, and negatively intervening) may not capture all possible ways of intervening behaviors in a bullying scenario. Also, among all the possible consequences of being bullied, we only adopted “fear of bullied” as the indicator. Although fear of being bullied has been identified as the most direct consequence of school bullying, other long-term consequences (such as depression and maladjustment) should further be incorporated in future research.

Finally, the current research adopted a non-anonymous survey format during data collection. Anonymous survey format is the preference for most existing studies on bullying, with the assumption that participants may reveal more truthful answers when personal information is not required. However, previous evidence on how anonymity might influence research validity is mixed. Some researchers propose that an anonymous survey could encourage participants to “exaggerate or make irresponsible responses” [38]. There is also evidence showing that results from anonymous and non-anonymous bullying surveys were not statistically different [39, 40]. O’Malley and colleagues [41] found that the assurance of confidentiality (but not anonymity) could be sufficient to obtain good validity, which was the practice in this study. Based on these results, the effects of anonymity could be mixed. Anonymity should be taken into consideration and multi-methods (i.e. peer-nomination, teacher assessment) should be used to evaluate bullying experiences in future studies.

## Conclusions

Our research discovered that family household demographic characteristics were related to the constructs of adolescents’ bullying at school. These risk characteristics can be used to inform practical guidance for school consolers about which students are at the higher risk of being involved in bullying and which students are at risk from suffering from negative consequences of being bullied. In particular, although abundant evidence has shown that children with or without siblings behave in many different ways [37], being an only child was not associated with any bullying-related constructs in the current research. Future research efforts are required to explore the relations between being an only child and bullying at school, with specific attention paid to generational changes that will result from the two, and now three-child policy in China.

## Supplementary Information

**Additional file 1.**

## Data Availability

The datasets used during the current study is available from the corresponding author on reasonable request.
