# Supplementary material for: Structural family factors and bullying at school: a large scale investigation based on a Chinese adolescent sample
Source: BMC Public Health. 2021 Dec 11;21:2249. doi: 10.1186/s12889-021-12367-3 (PMC8665508; doi:10.1186/s12889-021-12367-3)
Supplement: Supplementary file 1 — Additional file 1. [file 12889_2021_12367_MOESM1_ESM.docx]

**Dear all*

*We are a research team from XXX hospital. It’s a great pleasure for us to have you participate in this research. Bullying on campus is raising increasing concerns, and you may have been familiar with the phenomenon of bullying. The following questions were used to investigate bullying behaviors in your class. There’s no right or wrong for each question, and your response will be strictly kept anonymous. Please give your answer based on your observations. PLEASE NOTE: In this questionnaire, bullying refers to verbal insult (like teasing) or physical assaults (like pushing, shoving, kicking, slapping or hitting).*

Q1. During this school year how many times have you been bullied on school property?

□ Never.

□ Sometimes (1 or 2 times a month or more).

□ Often (1 or 2 times a week or more)

Q2. How many times in the past year (the last 12 months) have you bullied someone on school property?

□ Never.

□ Sometimes (1 or 2 times a month or more).

□ Often (1 or 2 times a week or more)

Q3. How many times in the past year (the last 12 months) have you been afraid of being bullied by someone on school property?

□ Never.

□ Sometimes (1 or 2 times a month or more).

□ Often (1 or 2 times a week or more)

Q4. How many times in the past year (the last 12 months) have you witnessed others being bullied on school property?

□ Never.

□ Sometimes (1 or 2 times a month or more).

□ Often (1 or 2 times a week or more)

Q5. 6. If you have saw bullying at school, what would you do?

□ I haven’t seen any bullying.

□ Ignored it as none of my business.

□ Nothing, just watched.

□ Joined in on the fun.

□ Tried to stop the bully or help the victim.

□ Asked for help from an adult or other student.

* Bullying-related variables used in the current research were shown above. We are really sorry that the complete version of the questionnaire could not be uploaded due to some copyright issues.
